# Interleukin-19 Gene-Deficient Mice Promote Liver Fibrosis via Enhanced TGF-β Signaling, and the Interleukin-19-CCL2 Axis Is Important in the Direction of Liver Fibrosis

**DOI:** 10.3390/biomedicines11072064

**Published:** 2023-07-22

**Authors:** Naoshige Ono, Takashi Fujita, Mariko Miki, Kazuhiro Nishiyama, Takeshi Izawa, Tomoko Aoyama, Mitsuru Kuwamura, Hideki Fujii, Yasu-Taka Azuma

**Affiliations:** 1 Laboratory of Prophylactic Pharmacology, Osaka Metropolitan University Graduate School of Veterinary Science, Osaka 598-8531, Japan; su23538q@st.omu.ac.jp (N.O.); sxc01037@edu.osakafu-u.ac.jp (M.M.); knishiyama@omu.ac.jp (K.N.); 2 Molecular Toxicology Laboratory, Department of Pharmaceutical Sciences, Ritsumeikan University, Shiga 525-8577, Japan; fujitat@fc.ritsumei.ac.jp (T.F.); 723tomoko@gmail.com (T.A.); 3 Laboratory of Veterinary Pathology, Osaka Metropolitan University Graduate School of Veterinary Science, Osaka 598-8531, Japan; takeshi.izawa@omu.ac.jp (T.I.); kuwamura@omu.ac.jp (M.K.); 4 Department of Hepatology, Graduate School of Medicine, Osaka Metropolitan University, Osaka 545-8585, Japan; fujiirola@yahoo.co.jp

**Keywords:** IL-19, liver, fibrosis, hepatic stellate cells, CCL2

## Abstract

IL-19 is a cytokine discovered by homologous searching with IL-10 and is produced by non-immune cells, such as keratinocytes, in addition to immune cells, such as macrophages. Liver fibrosis results from the inflammation and activation of hepatic stellate cells via chronic liver injury. However, the participation of IL-19 in liver fibrosis remains to be sufficiently elucidated. Our group studied the immunological function of IL-19 in a mouse model of carbon tetrachloride (CCl_4_)-induced liver fibrosis. IL-19 gene-deficient (KO) mice and body weight-matched wild-type (WT) mice were used. A liver fibrosis mouse model was created via CCl_4_ administration (two times per week) for 8 weeks. In CCl_4_-induced liver fibrosis, serum analysis revealed that IL-19 KO mice had higher ALT levels compared to WT mice. IL-19 KO mice had worse fibrosis, as assessed by morphological evaluation of total area stained positive with Azan and Masson trichrome. In addition, the expression of α-SMA was increased in liver tissues of IL-19 KO mice compared to WT mice. Furthermore, mRNA expression levels of TGF-β and α-SMA were enhanced in IL-19 KO mice compared to WT mice. In vitro assays revealed that IL-19-high expressing RAW264.7 cells inhibited the migration of NIH3T3 cells via the inhibited expression of CCL2 in the presence of CCl_4_ and IL-4. These findings indicate that IL-19 plays a critical role in liver fibrosis by affecting TGF-β signaling and the migration of hepatic stellate cells during liver injury. Enhancement of the IL-19 signaling pathway is a potential treatment for liver fibrosis.

## 1. Introduction

Interleukin (IL)-19 is a cytokine classified in the IL-10 family and is released from many immune and non-immune cells [1]. Major producing cells include macrophages, keratinocytes, epithelial cells, and vascular smooth muscle cells [2,3,4,5,6]. Interestingly, the IL-10 family, including IL-19, appeared evolutionarily earlier than the acquired immune system [7]. The IL-10 family, including IL-19, can promote innate immune responses, especially from epithelial cells, and control bacterial and viral infections in various infectious diseases [7]. We have previously shown, using IL-19 gene-deficient (KO) mice, that IL-19 plays a protective role in inflammatory bowel disease [1,2] and dermatitis [1]. Recently, we analyzed a mouse model of nonalcoholic steatohepatitis (NASH) induced by a NASH-induced diet and reported that liver fibrosis was worse in IL-19 KO mice compared to wild-type (WT) mice [8]. In NASH, fibrosis is formed by the involvement of inflammation in addition to a nonalcoholic fatty liver (NAFL) [9]. Nonalcoholic fatty liver disease (NAFLD) is a disease that includes NAFL and NASH. Obesity is the most relevant factor in the evolution of NAFLD [10]. Triglyceride (TG) accumulation occurs in the evolution of NAFL. In a previous study, we showed that IL-19 is involved in lipid metabolism [8]. In the same study, IL-19 inhibited TGs and cholesterols synthesis and reduced fatty liver [8]. These results elucidated some of the mechanisms by which liver fibrosis is exacerbated in IL-19KO mice. However, the action of IL-19 on the progression of fibrosis itself was not clear. In this study, we analyzed a mouse model of carbon tetrachloride (CCl_4_)-induced liver fibrosis against IL-19 KO mice. Right from the start, we obtained that CCl_4_-induced fibrosis was worse in IL-19 KO mice through increased expression of α-SMA and TGF-β, which are involved in fibrosis progression. In addition, we analyzed the mechanism of action of IL-19 and showed that IL-19 acts on Kupffer cells to produce CCL2, which causes hepatic stellate cells (HSCs) and myofibroblasts to migrate, and may be involved in the enhancement of fibrosis and the direction of fibrosis.

## 2. Materials and Methods

### 2.1. Mice

IL-19 KO mice on a BALB/c background used in the past were used. Mice lacking the IL-19 gene in one allele were crossed to obtain KO mice lacking IL-19 from both alleles and WT mice with the IL-19 gene in both alleles. Mice 7–8 weeks old were used, and all sexes were male. The method procedures with animals implemented in this study were approved by the Osaka Prefecture University Animal Care and Committee. All procedures used in this study complied with institutional policies of the Osaka Prefecture University Animal Care and Use Committee.

### 2.2. Fibrosis Induction

Liver fibrosis was induced by repeated injection of CCl_4_. CCl_4_ (Sigma-Aldrich, Saint Louis, MI, USA) was diluted in olive oil (Sigma-Aldrich) to 50% and injected into mice at a dose of 1 mL/kg of CCl_4_. Mice were administered an i.p. injection using a syringe of insulin 1 mL × 29 G (Terumo Co., Tokyo, Japan) with either olive oil or CCl_4_ twice a week for 8 weeks. Three days after the final CCl_4_ injection, all mice were euthanized via isoflurane anesthesia. The weights of the removed livers were first recorded. Blood was taken from the heart. We performed three independent experiments of fibrosis induction with CCl_4_ injection. The number of examples in one independent experiment was then tested using four or more examples.

### 2.3. Aminotransferase Activities

After serum was prepared from the blood samples, the level of the enzymes alanine aminotransferase (ALT) and aspartate aminotransferase (AST) was calculated during the day using a Transaminase CII test WAKO (FUJIFILM Wako Pure Chemical, Osaka, Japan).

### 2.4. Histological Evaluation of the Liver and Immunohistochemical Staining

The explanted liver was soaked with 10% neutral buffered formalin and embedded in paraffin. Hematoxylin and eosin (H&E) staining was applied for histological evaluation. Azan and Masson trichrome staining were performed to assess hepatic fibrosis [8]. For Masson trichrome staining, the sections were immersed overnight in 50% potassium dichromate, stained with hematoxylin, and incubated in Ponceau S dye. The sections were then washed and incubated with 1% phosphomolybdic acid prior staining with aniline blue. 

Immunohistochemical staining for paraffin-embedded sections was performed as described previously [8]. Paraffin-embedded sections (3 μm thick) were stained with anti-α-SMA antibody as a marker of myofibroblasts. The immunoreactivity of α-SMA was detected using the horseradish peroxidase-3,3-diaminobenzidine (DAB) system. Images were taken with a VS120 Virtual Slide Scanner (Olympus Corporation, Tokyo, Japan). Fibrotic area and α-SMA-positive area were assessed via quantification using Image J. The results were expressed as a percentage of positive area per total area. Three to four views per sample were evaluated.

### 2.5. Immunofluorescent Staining

Immunofluorescent staining for frozen sections was performed as described previously, with some modifications. Briefly, mice were fixed by transcardiac perfusion with 4% paraformaldehyde, and the liver was isolated. Frozen sections (5 μm thick) were stained with anti-F4/80 mouse monoclonal antibody (1:400) (TONBO biosciences, San Diego, CA, USA) and anti-mouse IgG Alexa Fluor 568 conjugated secondary antibody, anti-IL-19 rabbit polyclonal antibody (1:200) (Abcam, Cambridge, UK) and anti-rabbit IgG Alexa Fluor 488 conjugated secondary antibody, and DAPI (4′,6-diamidino-2-phenylindole dihydrochloride) for nuclear staining. Fluorescence images were captured using a VS120 Virtual Slide Scanner (Olympus Corporation).

Immunofluorescent staining for paraffin-embedded sections was performed as described previously, with minor modifications [10]. For characterization of HSCs, paraffin-embedded sections (3 μm thick) were stained with PE-conjugated anti-Ly-6A/E (Sca-1) antibody (1:200) (108-108, Biolegend, San Diego, CA, USA), anti-Ki67/MKI67 antibody (SP6) (1:500) (NB600-1252, Novus Biologicals, Centennial, CO, USA), and anti-mouse IgG Alexa Fluor 488 conjugated secondary antibody. For characterization of M2 macrophage, sections were stained with PE-conjugated CD163 antibody (155307) (1:200) (Biolegend). Fluorescence images were taken using microscopy (BX51/DP74) and cellSens software ver. 3.2 (Olympus, Tokyo, Japan).

Positive area was assessed by quantification using Image J. The results were expressed as a percentage of positive area per total area. Four to five views per sample were evaluated.

### 2.6. RNA Separation and Quantitative Real-Time PCR (QPCR)

Liver samples or isolated cells were homogenized. Total RNA was separated by Sepasol (Nacalai Tesque, Kyoto, Japan), and separated total RNA was used to synthesize complementary DNA using SuperScript Reverse Transcriptase (Roche, Madison, WI, USA) [8]. QPCR analysis was performed by SYBR Green (Toyobo, Osaka, Japan) [8]. Supplementary Table S1 contains the sequences of the primers used. Hprt or Gapdh was used as the internal standard. 

### 2.7. In Vitro Cell Culture

NIH3T3 cells were obtained from Riken Cell BANK (Ibaraki, Japan) and maintained in DMEM with 10% FBS and antibiotics. RAW264.7 and its clones were cultured in 10% FCS-DMEM. 

### 2.8. IL-19-High Expressing RAW264.7

Synthetic nucleotides for murine IL-19 (NM_001009940) were subcloned into pMXs-puro retrovirus vector, and gene transfer was carried out using PLAT-E packaging cells. Established MOCK/RAW264.7 or IL-19/RAW264.7 cells were stored in a deep freezer until use. To mimic CCl_4_ treatment in vivo, RAW264.7 and NIH3T3 cells were treated with 1 mM CCl_4_. IL-4 (10 ng/mL) was added to the RAW264.7 cells to make the M2 phenotype.

### 2.9. Chemotaxis Assay

Chemotaxis assay was performed using Boyden chamber assay (Neuroprobe, Cabin John, MD, USA). Briefly, the supernatants from RAW264.7 cells treated with or without CCl_4_ were plated in the lower component (50 μL), and NIH3T3 cells treated with or without CCl_4_ were plated in the upper component (200 μL). After 12 h incubation, a PVP-free Nuclepore membrane (8 μm) was fixed with methanol and stained with Diff-Quick (Sysmex Corp., Hyogo, Japan). The upper side of the filter was then scraped free of cells. The absorbance of the membrane that migrated NIH3T3 cells to the lower side was determined. 

### 2.10. Statistical Analysis

Liver weight, ALT/AST quantification, F4/80-positive area, and chemotaxis, as a first step, were performed using one-way ANOVA analysis for non-repeated measures to detect differences among groups. The differences between groups were determined using the Tukey–Kramer test. The differences in the survival rate were evaluated using the Kaplan–Meier test. Other data were evaluated using the two-tailed Student’s *t*-test (unpaired) to detect differences between 2 groups. A *p* value of less than 0.05 was considered statistically significant. 

## 3. Results

### 3.1. Survival and Body and Liver Weights

**Figure 1:**
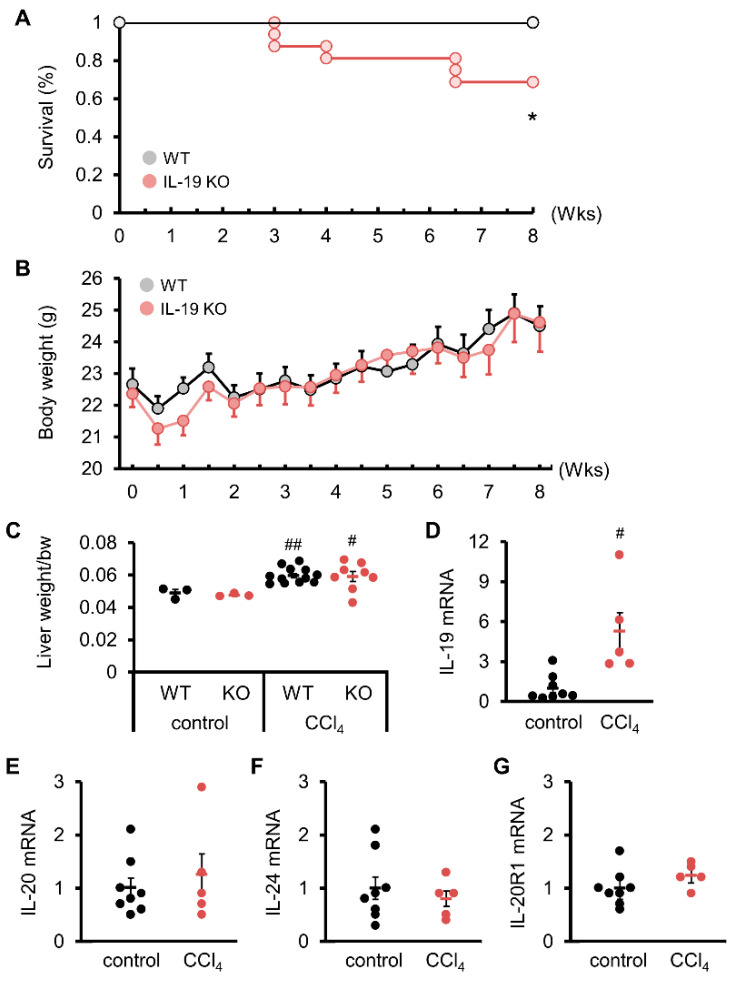
Induction of fibrosis reduced survival without affecting the body weight or liver weight of IL-19 KO mice. WT and IL-19 KO mice were treated with CCl_4_ twice a week for 8 weeks. Survival (*n* = 11–16) (**A**) and body weight (*n* = 16) (**B**) were monitored twice a week. * *p* < 0.05 vs. WT. (**C**) WT and IL-19 KO mice were treated with either olive oil (*n* = 3) (control) or CCl_4_ (*n* = 8–12) twice a week for 8 weeks. Liver weight was measured. ^#^ *p* < 0.05, ^##^ *p* < 0.01 vs. each control. (**D**–**G**) mRNA expressions in the livers of WT mice treated with either olive oil (*n* = 8) (control) or CCl_4_ (*n* = 5) twice a week for 8 weeks. ^#^ *p* < 0.05 vs. control.

We started the experiment and noticed a difference in survival rates. All WT mice survived upon CCl_4_ administration (Figure 1A). More than 30% of the IL-19KO mice died, showing a significantly lower survival rate than WT mice (Figure 1A). Upon CCl_4_ administration, the body weight of WT and IL-19 KO mice showed an increase over time, with no significant differences between the two groups (Figure 1B). Both WT and IL-19 KO mice upon CCl_4_ administration had significantly increased liver weight than those upon vehicle administration (control) (Figure 1C). However, liver weight was similar in WT and IL-19 KO mice upon CCl_4_ administration. The expression of IL-19 in WT mice treated with CCl_4_ for 8 weeks was analyzed. The QPCR results showed that CCl_4_ administration significantly enhanced the level of IL-19 in WT mice compared to the controls (Figure 1D). IL-19 KO mice can have an altered balance of several other cytokines due to the KO of IL-19. It is important to measure the levels of IL-20 and IL-24, which use the same receptors as IL-19. We confirmed that there were no expression changes associated with liver fibrosis in the mRNA expression levels of IL-20, IL-24, and IL-20R1 (Figure 1E–G).

### 3.2. ALT and AST

**Figure 2:**
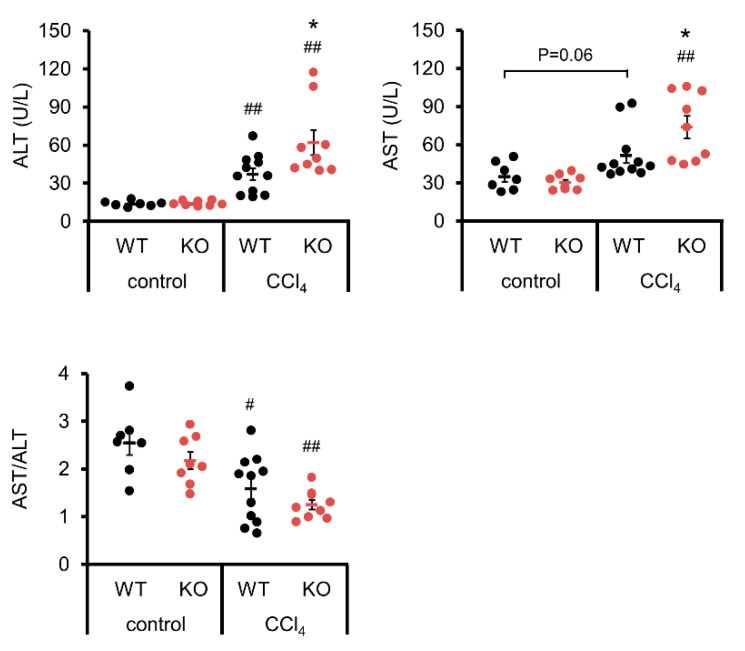
Induction of fibrosis increased ALT and AST levels in IL-19 KO mice. WT and IL-19 KO mice were treated with either olive oil (*n* = 7–8) (control) or CCl_4_ (*n* = 9–11) twice a week for 8 weeks. Serum ALT and AST levels were measured, and the AST/ALT ratio was calculated. * *p* < 0.05 vs. WT in CCl4. ^#^ *p* < 0.05, ^##^ *p* < 0.01 vs. each control.

Blood tests for activities of enzymes such as ALT and AST can be used to examine the liver function. ALT was significantly enhanced in WT and IL-19 KO mice upon CCl_4_ administration compared to those upon vehicle control (Figure 2). AST was significantly enhanced in IL-19 KO mice, but not WT mice, upon CCl_4_ administration compared to those upon vehicle control (Figure 2). Upon CCl_4_ administration, both ALT and AST in IL-19 KO mice were worsened compared to WT mice. The AST/ALT ratio was calculated. The AST/ALT ratio decreased significantly with liver fibrosis, but there was no significant difference between WT and IL-19KO mice (Figure 2). 

### 3.3. Liver Histology

**Figure 3:**
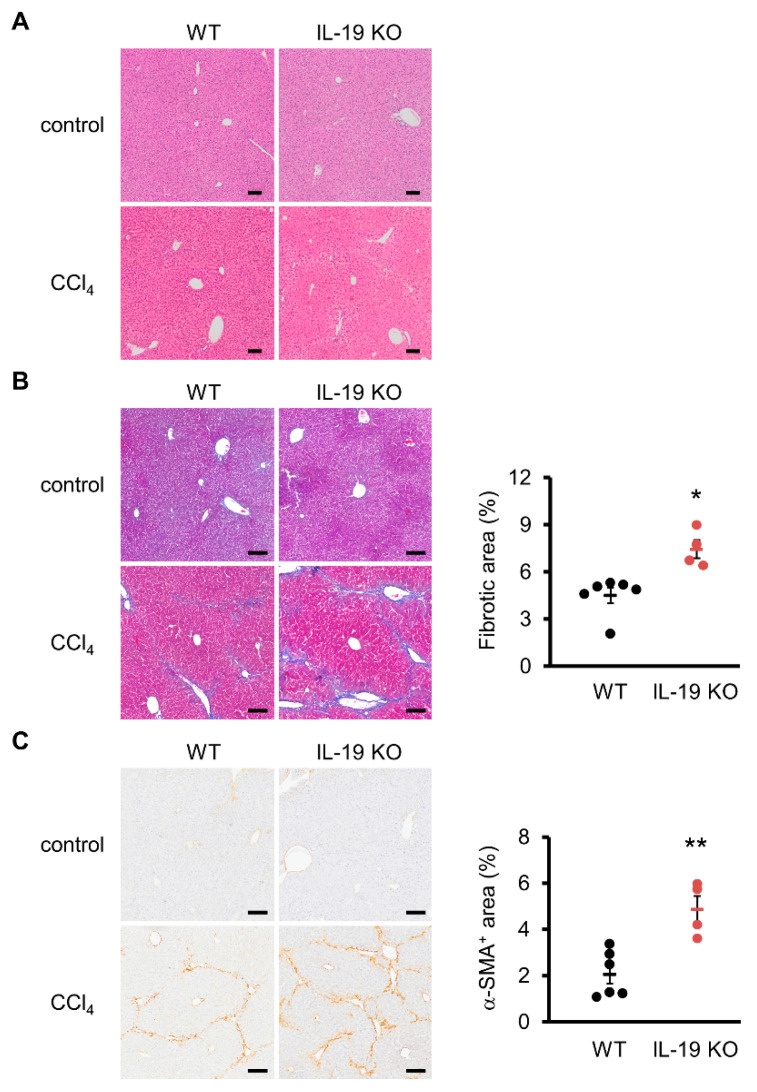
Induction of fibrosis increased fibrosis and α-SMA-positive areas in IL-19 KO mice. WT and IL-19 KO mice were treated with either olive oil (*n* = 3) (control) or CCl_4_ (*n* = 4–6) twice a week for 8 weeks. (**A**) Representative liver sections stained with H&E are shown. (**B**) Representative liver sections stained with Azan staining are shown. The blue color was quantified using ImageJ and evaluated as fibrosis. (**C**) Representative liver sections immunohistochemically stained with α-SMA are shown. Positive area was quantified using ImageJ. Positive area was expressed as % per 0.6 mm^2^. Scale bars are 100 μm. * *p* < 0.05, ** *p* < 0.01 vs. WT.

Histopathological analysis with H&E and Azan staining showed that WT mice, upon CCl_4_ administration, experienced hepatocellular hypertrophy with karyomegaly, dystrophic calcification in necrotized hepatocytes, and mild inflammatory cell infiltrate in the centrilobular region with mild bridging fibrosis (Figure 3A,B). In IL-19 KO mice, upon CCl_4_ administration, the histopathological lesions became more prominent; particularly, there was marked hepatocellular degeneration (karyomegaly and vacuolar degeneration), necrosis, and inflammatory cell infiltrate in the centrilobular hepatocytes in the centrilobular region, associated with moderate to extensive bridging fibrosis (Figure 3A,B). The fibrosis area was greater in IL-19 KO mice than in WT mice (Figure 3B).

Collagen-producing cells were quantified via immunostaining with α-SMA, a myofibroblast marker. There was a significant increase in α-SMA-positive area in IL-19 KO mice with CCl_4_ administration, compared with WT mice with CCl_4_ administration (Figure 3C). 

### 3.4. Factors Involved in Fibrosis Progression

**Figure 4:**
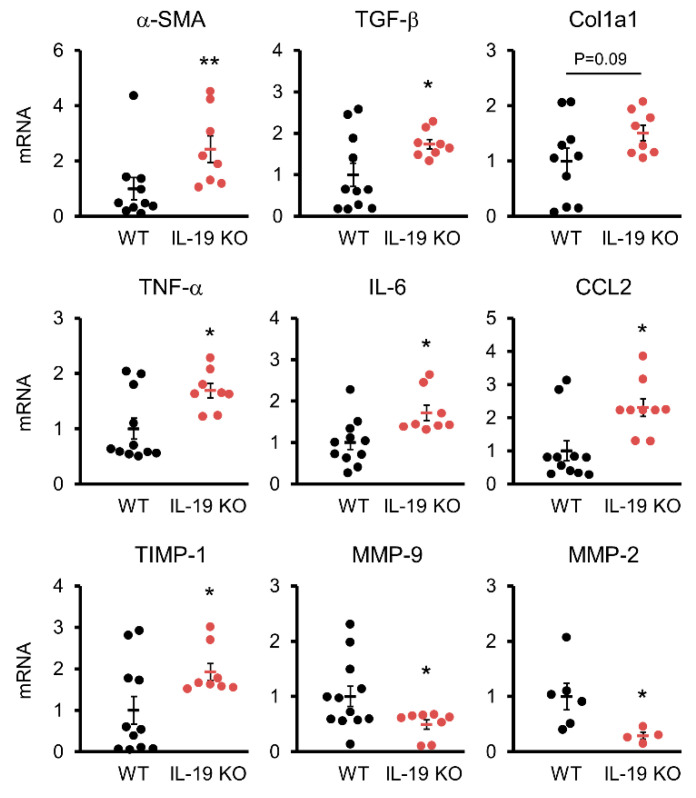
Variations in factors involved in fibrosis. mRNA expressions of several factors in the livers of WT (*n* = 6–12) and IL-19 KO (*n* = 4–8) mice that were treated with CCl_4_ twice a week for 8 weeks. * *p* < 0.05, ** *p* < 0.01 vs. WT.

We analyzed factors involved in the generation and degradation of fibrosis and inflammation in WT and IL-19 KO mice upon CCl_4_ administration. TGF-β, a factor involved in fibrosis, was measured. QPCR results showed significantly increased expression of α-SMA and TGF-β in IL-19 KO mice compared to WT mice (top of Figure 4). Col1a1 expression was enhanced in IL-19 KO mice compared to WT mice, but not significantly. In the liver, inflammation within a tissue is the trigger for fibrosis development. IL-6, TNF-α, and CCL2, factors involved in inflammation, were measured. These factors were significantly enhanced in IL-19 KO mice compared to WT mice (middle of Figure 4). Matrix metalloproteinases (MMPs) are involved in the extracellular degradation system of collagen. On the other hand, the tissue inhibitors of matrix metalloproteinases (TIMPs) act as endogenous MMP inhibitors and regulate collagen degradation. Interestingly, TIMP-1 was significantly enhanced in IL-19 KO mice compared to WT mice, whereas MMP-2 and MMP-9 were significantly decreased (lower Figure 4).

### 3.5. IL-19 Location in the Liver

**Figure 5:**
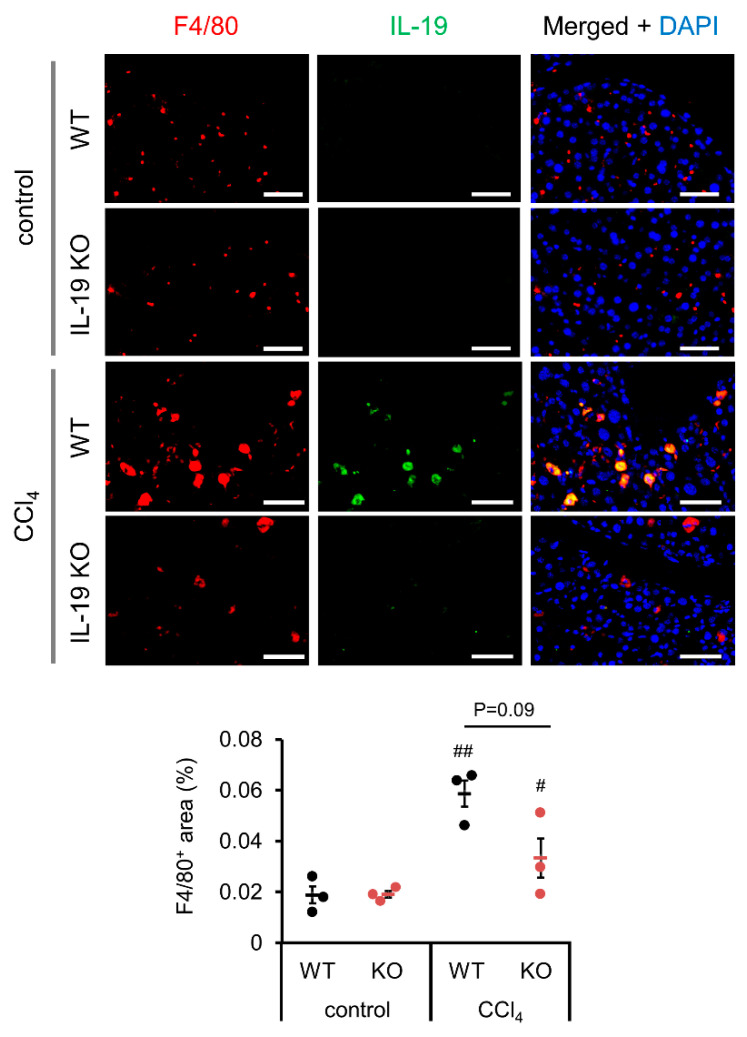
IL-19 expression is upregulated by fibrosis induction and F4/80-positive cells express IL-19. WT and IL-19 KO mice were treated with either olive oil (control) or CCl_4_ twice a week for 8 weeks. Representative liver sections’ immunofluorescence stainings with IL-19 and F4/80 antibodies with DAPI are shown (*n* = 3). Scale bar is 50 μm. F4/80-positive area was quantified using ImageJ. Positive area was expressed as % per 0.045 mm^2^. ^#^ *p* < 0.05, ^##^ *p* < 0.01 vs. each control.

We investigated the location of IL-19-producing cells in the liver. The Kupffer cell marker F4/80 and IL-19 were co-immunostained since we know from some reports that IL-19 was released from macrophages [2] and microglia [1]. As shown in the upper panels, little IL-19 expression was observed in the livers from untreated WT mice (Figure 5). As shown in the lower panels, IL-19 expression was observed following CCl_4_ administration to WT mice, and the IL-19-expressing cells were F4/80-positive Kupffer cells (Figure 5). As shown in the IL-19 KO panels, IL-19 expression is absent in IL-19KO mice (Figure 5). Subsequently, F4/80-positive cells were quantified as well. F4/80-positive cells appeared to be reduced with IL-19 KO mice, but the difference was not significant (Figure 5). 

### 3.6. Possible Action of IL-19 on Fibrosis Progression

**Figure 6:**
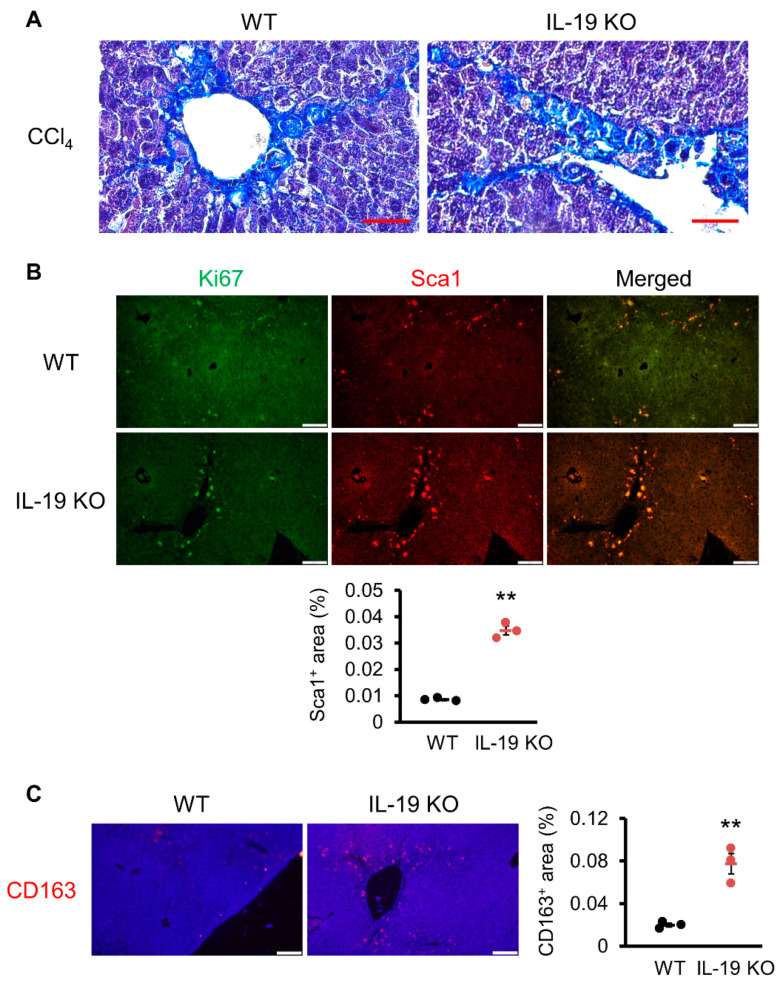
Induction of fibrosis increased proliferative cells and M2 macrophages in IL-19 KO mice. WT and IL-19 KO mice were treated with CCl_4_ (*n* = 4–6) twice a week for 8 weeks. (**A**) Representative liver sections stained with Masson trichrome staining are shown (*n* = 3). Scale bar is 50 μm. (**B**) Representative liver sections immunofluorescence staining with Ki67 and Sca1 antibodies are shown (*n* = 3). Scale bar is 100 μm. Sca1-positive area was quantified using ImageJ. Positive area was expressed as % per 0.45 mm^2^. ** *p* < 0.01 vs. WT. (**C**) Representative liver sections’ immunofluorescence stainings with CD163 antibody are shown (*n* = 3). Scale bar is 100 μm. CD-163-positive area was quantified using ImageJ. Positive area was expressed as % per 0.45 mm^2^. ** *p* < 0.01 vs. WT.

Analysis using a mouse model revealed that liver fibrosis is worse in IL-19 KO mice. Further experiments were conducted to clarify the mechanism of action of IL-19 at the cellular level. Figure 6A shows the results of the Masson trichrome staining. Masson trichrome staining, like Asan staining, stains collagen fibers, but also stains nuclei, providing more information. Histopathological analysis with Masson trichrome staining showed that myofibroblasts cells, whose nuclei are stained black, are scattered along fibers that are stained blue. Next, we analyzed the localization of HSCs involved in increased fibrosis progression. Sca1, as a marker of HSCs, was analyzed for co-localization with Ki67, an indicator of cell proliferative activity. In IL-19 KO mice, more Sca1-positive HSCs were found in the perivascular area than in WT mice, and they were co-localized with Ki67 (Figure 6B). In Figure 6C, we examined the localization of CD163, a M2 macrophage marker. Significantly, IL-19 KO mice showed a clear increase in CD163-positive cells. These results in Figure 6 show that there is an increase in M2 macrophages in IL-19 KO mice and also indicate that HSCs develop fibrosis while proliferating in IL-19 KO mice. 

### 3.7. Alteration in Chemotaxis Using NIH3T3 Cells and High-IL-19-Expressing RAW264.7 Cells

**Figure 7:**
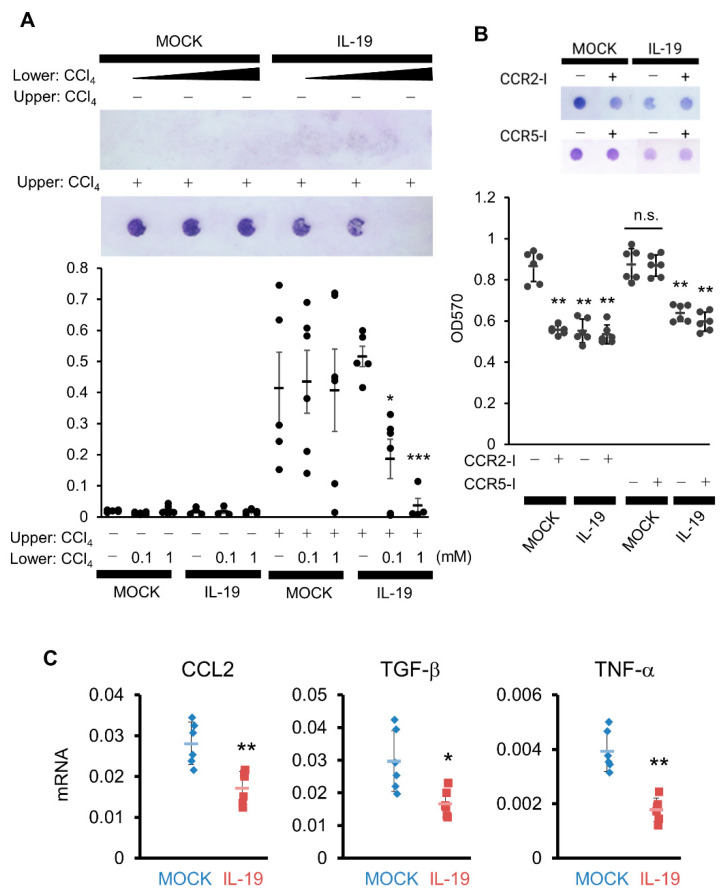
Overexpression of IL-19 on macrophages inhibits the migration of NIH3T3 cells. (**A**) NIH3T3 cells treated with or without CCl_4_ were placed in the upper cell, with the culture supernatant from MOCK or IL-19-high expressing RAW264.7 cells treated with or without CCl_4_ (1 mM) in the lower well, followed by incubation for 12 h (*n* = 5). Cells stained with circles indicate migrated cells. * *p* < 0.05, *** *p* < 0.001 vs. without CCl_4_. (**B**) NIH3T3 cells treated with CCl_4_ (1 mM) in the presence of CCR2 inhibitor, BMS CCR2 22 (100 nM), or CCR5 inhibitor, TAK779 (100 nM), were placed in the upper cell, with the culture supernatant from MOCK or IL-19-high expressing RAW264.7 cells treated with CCl_4_ in the lower well, followed by incubation for 12 h (*n* = 4). Cells stained with circles indicate migrated cells. ** *p* < 0.01 vs. without inhibitor (MOCK). (**C**) MOCK or IL-19-high expressing RAW264.7 cells were treated with CCl_4_ in the presence of IL-4 for 24 h. mRNA expressions of CCL2, TGF-β, and TNF-α in the cells were calculated by QPCR (*n* = 6). * *p* < 0.05, ** *p* < 0.01 vs. MOCK.

From the results in Figure 6, it is possible that HSCs cells present in the space of Disse are migrating and causing bridging fibrosis. Given chemokine secretion, Kupffer cells and/or macrophages are likely the first targets. We performed an in vitro chemotaxis assay using NIH3T3 and IL-19-high expressing RAW264.7 cells as substitutes for HSCs and Kupffer cells/macrophages. Under conditions where there is no CCl_4_, no cell migration occurred when a culture supernatant of high-IL-19-expressing RAW264.7 cells was placed in the lower component and NIH3T3 cells were placed in the upper component (Figure 7A left side white columns). Importantly, the CCl_4_ treatment of NIH3T3 cells resulted in increased migration, and this increased migration was significantly suppressed when culture supernatant of IL-19-high expressing RAW264.7 cells by CCl_4_ treatment was placed lower (Figure 7A right side black columns). Thus, RAW264.7 cells, assuming Kupffer cells/macrophages, in contact with both CCl_4_ and IL-19 may suppress the production of chemokines involved in the migration of NIH3T3 cells, assuming HSCs. 

CCL2 and CCL3 are assumed to be chemokines that migrate HSCs and myofibroblasts. We evaluated chemotaxis using inhibitors of CCR2, the receptor for CCL2, and CCR5, the receptor for CCL3. The addition of a CCR2 inhibitor (CCR2-I) to MOCK/RAW264.7 cells significantly inhibited migration in NIH3T3 cells by CCl_4_, comparable to the inhibition achieved with the high expression of IL-19 (Figure 7B). The inhibition of migration in NIH3T3 cells with the high expression of IL-19was not further suppressed by the addition of CCR2-I (Figure 7B). In contrast, the addition of a CCR5 inhibitor (CCR5-I) did not inhibit migration in NIH3T3 cells (Figure 7B). Therefore, CCL2 is likely involved in the inhibition of migration in NIH3T3 cells by high-IL-19-expressing RAW264.7 cells.

We then measured the production of CCL2 as chemokines that migrate HSCs or myofibroblasts. When CCL2 expression was quantified by QPCR after CCl_4_ treatment of MOCK-expressing or high-IL-19-expressing RAW264.7 cells under conditions in which IL-4 was added to induce the M2 condition, there was significantly decreased expression of CCL2 in the high-IL-19-expressing RAW264.7 cells (Figure 7C). In experiments under the same conditions, TGF-β and TNF-α expressions were also significantly suppressed in high-IL-19-expressing RAW264.7 cells (Figure 7C).

## 4. Discussion

CCl_4_ is a typical drug that induces liver damage, and repeated administration of CCl_4_ results in fibrosis of the liver [11]. After repeated administration of CCl_4_, WT mice were weakly and mildly fibrotic with increased liver weight and elevated ALT. A noteworthy result was that IL-19 KO mice showed clear fibrosis with increased liver weight and increased ALT and AST. Body weights were similar between WT and IL-19 KO mice. Therefore, differences in the degree of fibrosis may not be reflected in differences in liver weight. ALT and AST were higher in IL-19 KO mice than in WT mice. In inactive chronic hepatitis, ALT levels are higher. In acute hepatitis, there is a rapid rise in AST levels and rapid necrosis of hepatocytes. ALT and AST levels also increase with the onset of fibrosis [12]. Therefore, since ALT and AST are elevated in IL-19 KO mice, it is reasonable to suggest that tissue damage was worse in the IL-19 KO mice compared to WT mice.

Liver fibrosis occurs when HSCs, which normally store vitamin A, differentiate into myofibroblasts and secrete collagens, including Col1a1, abnormally upon various stimuli [13]. As a result, hepatocytes responsible for liver function are replaced by extracellular matrices, including Col1a1. Since α-SMA-positive cells are generated during the differentiation of HSCs into myofibroblasts, α-SMA is used as an indicator of collagen production and fibrosis [14]. Since TGF-β promotes the differentiation of HSCs into myofibroblasts, TGF-β is also used as an indicator of fibrosis [15]. Consistent with the Azan staining, Masson trichrome staining, and immunohistochemical staining results, α-SMA and TGF-β expressions were higher in IL-19 KO mice according to QPCR. It is clear that fibrosis is developed by IL-19 KO mice. As fibrosis progresses, type IV collagens in addition to type I collagens increase [16]. Recently, type IV collagen has been recognized as a useful indicator of liver fibrosis because it is recognizable early in the process and also reflects the progression of fibrosis [17,18]. MMPs degrade collagens and other extracellular matrices [19]. MMPs are induced by IL-1, TNF-α, EGF, PDGF, and FGF [19,20,21,22], while their activity is inhibited by an inhibitor, TIMP [23]. MMP2 is mainly produced by fibroblasts [24], while MMP9 is mainly produced by macrophages [25]. TIMPs, inhibitors of MMPs, regulate the balance between ECM degradation and synthesis by MMPs [26]. Decreased MMP-2 and MMP-9 and increased TIMP-1 were found as part of the mechanism of developed fibrosis with IL-19 KO. In IL-19 KO, the increase in TIMP-1 may have inhibited the activity of MMPs and promoted the accumulation of fibrosis. A reduction in survival was also observed in IL-19 KO. The primary mechanism of CCl_4_ is damage to liver hepatocytes. Therefore, we believe that CCl_4_ administration increased the rate of liver hepatocytes damage and fibrosis in the liver, which led to death due to liver dysfunction. Further analysis is needed to determine whether only worsening liver fibrosis can explain individual deaths.

The activation of HSCs occurs because myofibroblasts produce collagens and contribute to fibrosis progression. This expectation was supported by the increased number of Sca1-positive and Ki67-positive cells in the IL-19 KO mice compared to WT mice. Interestingly, M2 macrophages were increased in IL-19 KO. M2 macrophages are known to be involved in fibrosis and tissue repair. Furthermore, high-IL-19-expressing macrophages inhibited the expression of CCL2, a chemokine that migrates HSCs and myofibroblasts. Figure 4 showed that CCL2 expression was increased in the liver of IL-19 KO mice. Increased CCL2 expression in IL-19 KO mice may have contributed to increased fibrosis. Moreover, it is important to note that suppression by IL-19 was observed under CCl_4_ treatment and M2 conditions. Thus, the function of IL-19 in the liver may be to inhibit fibrosis progression by inhibiting the migration of HSCs and myofibroblasts. Moreover, IL-19 inhibited TNF-α and TGF-β expression from macrophage lineage cells in an in vitro assay. Thus, even at the factor level, IL-19 plays an inhibitory role in the development of inflammation and fibrosis. This result is supported by the in vivo QPCR results. TGF-β is still important in fibrosis. TGF-β is the most well-known factor in the progression of liver fibrosis [27]. HSCs are noted for fibrosis progression because of their role in making collagen and other extracellular matrices [28]. TGF-β is a potent inducer of HSCs and stimulates collagen production [29]. Two types of macrophage-based cells are present in the liver: Kupffer cells, which are a type of tissue macrophage specific to the liver, and bone-marrow-derived macrophages that infiltrate in response to inflammation or injury. Both resident Kupffer cells and bone-marrow-derived macrophages can activate HSCs because of their TGF-β producing ability [30]. On the other hand, TGF-β is also produced by hepatocytes in small amounts. Thus, any or all of Kupffer cells, bone-marrow-derived macrophages, and hepatocytes may be involved in the increased TGF-β production in IL-19 KO mice.

Another result showed that inflammatory cytokines were increased in IL-19 KO mice compared to in WT mice. Liver damage caused by, for example, CCl_4_ elicits an inflammatory response. Prolonged inflammation exacerbates liver damage, making fibrosis more developed. These results are also responsible for the worsening of fibrosis in IL-19 KO mice. Next, we focus on the variation in IL-6 and TNF-α. Both factors are produced primarily by Kupffer cells and bone-marrow-derived macrophages. The simplest consideration is that IL-6 and TNF-α act on hepatocytes to promote TGF-β production and then activate HSCs [31]. Similar to the TGF-β consideration, IL-6 and TNF-α production was increased from Kupffer cells and bone-marrow-derived macrophages in IL-19 KO mice. In a previous study, bone-marrow-derived macrophages from IL-19 KO mice had increased lipopolysaccharide-stimulated IL-6 and TNF-α production [2]. Thus, the loss of the IL-19 gene may increase inflammatory cytokine production from Kupffer cells and macrophages. As described above, we have identified a novel role for IL-19 in fibrosis progression. Future analysis should focus on the undocumented role of IL-19 on HSCs.

## 5. Conclusions

In this study, we showed that loss of the IL-19 gene exacerbated liver fibrosis. As a mechanism, IL-19 may act on Kupffer cells/macrophages to make chemokines and modulate the migration of HSCs towards chemokines and regulate differentiation into myofibroblasts via TGF-β. This is the first report that IL-19 KO mice exacerbated liver fibrosis. In conclusion, the role of IL-19 in liver fibrosis is now fairly clear, although it is not yet complete. It is clinically useful to show that supplying IL-19 may reduce liver fibrosis.

## Data Availability

Not applicable.

## Supplementary Materials

The following supporting information can be downloaded at: https://www.mdpi.com/article/10.3390/biomedicines11072064/s1, Table S1: Primers.

## Abbreviations

ALT	alanine aminotransferase
AST	aspartate aminotransferase
CCl_4_	carbon tetrachloride
DAB	3,3-diaminobenzidine
H&E	hematoxylin and eosin
HSCs	hepatic stellate cells
IL	interleukin
KO	gene-deficient
MMP	matrix metalloproteinases
NAFLD	nonalcoholic fatty liver disease
NAFL	nonalcoholic fatty liver
NASH	nonalcoholic steatohepatitis
QPCR	quantitative real-time PCR
TIMP	tissue inhibitor of matrix metalloproteinase
TG	triglyceride
WT	wild-type
