# Supplementary material for: Interleukin-19 Gene-Deficient Mice Promote Liver Fibrosis via Enhanced TGF-β Signaling, and the Interleukin-19-CCL2 Axis Is Important in the Direction of Liver Fibrosis
Source: Biomedicines. 2023 Jul 22;11(7):2064. doi: 10.3390/biomedicines11072064 (PMC10377488; doi:10.3390/biomedicines11072064)
Supplement: Supplementary file 1 [file biomedicines-11-02064-s001.zip › biomedicines-2414858-supplementary.pdf]

Supplementary table 1. Primers

gene	forward	reverse	accession number	product length (bp)
IL-19	CTCCTGGGCATGACGTTGATT	GCATGGCTCTCTTGATCTCGT	NM_001009940.2	118
IL-20	TCTTGCCTTTGGACTGTTCTCC	GTTTGCAGTAATCACACAGCTTC	NM_021380.2	100
IL-24	GAGTTGGGGACTACAGATTCTCC	GTGCACTCTCACTAATGGGAAGC	NM_053095.3	422
IL-20R1	CTAAGTCGAGAAGAACGTGGT	TGACTTTAGCCTTCCATGCTGA	NM_172786.2	195
α-SMA	GTCCCAGACATCAGGGAGTAA	TCGGATACTTCAGCGTCAGGA	NM_007392.3	102
TGF-β	TACAGGGCTTTCGATTCAGC	CGCACACAGCAGTTCTTCTC	NM_011577.2	247
Col1a1	GCTCCTCTTAGGGGCCACT	ATTGGGGACCCTTAGGCCAT	NM_007742.4	91
TNF-α	CATCTTCTCAAAATTCGAGTGACAA	TGGGAGTAGACAAGGTACAACCC	NM_013693.3	175
Il-6	CTGCAAGAGACTTCCATCCAG	AGTGGTATAGACAGGTCTGTTGG	NM_031168.2	131
CCL2	CCCAATGAGTAGGCTGGAGA	TCTGGACCCATTCCTTCTTG	NM_011333.3	125
TIMP-1	CTTGGTTCCCTGGCGTACTC	ACCTGATCCGTCCACAAACAG	NM_001044384.1	150
MMP-9	TGAATCATAGAGGAAGCCCATTACA	CGGAGTCCAGCGTTGCA	NM_013599.5	81
MMP-2	GTGTTCTTCGCAGGGAATGAG	GATGCTTCCAAACTTCACGCT	NM_008610.3	363
GAPDH	ACCCACTCCTCCACCTTTG	CTCTTGTGCTCTTGCTGGG	NM_001256799.3	178
HPRT	GTTGGATACAGGCCAGACTTTGTTG	GAGGGTAGGCTGGCCTATAGGCT	NM_013556.2	352
